# Immunodeficiency, HIV Viremia, and Incident Anal Cancer Among People With HIV in South Africa

**DOI:** 10.1093/ofid/ofaf693

**Published:** 2025-11-12

**Authors:** Nathalie V Fernández Villalobos, Yann Ruffieux, Chido Chinogurei, Andreas D Haas, Nicola Low, Matthias Egger, Jenni Noble, Naomi Folb, Gary Maartens, Eliane Rohner

**Affiliations:** Institute of Social and Preventive Medicine, University of Bern, Bern, Switzerland; Institute of Social and Preventive Medicine, University of Bern, Bern, Switzerland; Centre for Integrated Data and Epidemiological Research, School of Public Health, University of Cape Town, Cape Town, South Africa; Institute of Social and Preventive Medicine, University of Bern, Bern, Switzerland; Centre for Integrated Data and Epidemiological Research, School of Public Health, University of Cape Town, Cape Town, South Africa; Institute of Social and Preventive Medicine, University of Bern, Bern, Switzerland; Centre for Integrated Data and Epidemiological Research, School of Public Health, University of Cape Town, Cape Town, South Africa; Department of Infectious Diseases and Hospital Epidemiology, University Hospital Zurich, University of Zurich, Zurich, Switzerland; Population Health Sciences, Bristol Medical School, University of Bristol, Bristol, UK; Medscheme, Cape Town, South Africa; Medscheme, Cape Town, South Africa; Division of Clinical Pharmacology, Department of Medicine, University of Cape Town, Cape Town, South Africa; Division of Clinical Pharmacology, Department of Medicine, University of Cape Town, Cape Town, South Africa; Institute of Social and Preventive Medicine, University of Bern, Bern, Switzerland

**Keywords:** Anal cancer, HIV, immunodeficiency, viral load, CD4 cell count, South Africa

## Abstract

Among 130 992 people with HIV (PWH) in South Africa, 60 anal cancers were diagnosed. Lower CD4 counts and, to a lesser extent, higher HIV RNA viral loads were associated with an increased anal cancer risk. Maintaining high CD4 counts may contribute to anal cancer prevention among PWH in South Africa.

People with HIV (PWH) are at substantially higher risk of developing anal cancer than people without HIV [1, 2]. Most anal cancers are caused by persistent infection with high-risk genotypes of human papillomavirus (hrHPV), which drives the formation of high-grade squamous intraepithelial lesions (HSIL), the precursor of anal cancer. Persistent infection with hrHPV and progression to HSIL are more common among PWH than people without HIV [3].

The risk of developing anal cancer seems to be particularly elevated among individuals with low CD4 counts [4, 5] or AIDS [2]. Studies in North America and Europe have found nadir and lagged CD4 counts to be better predictors of anal cancer risk than current CD4 counts [5, 6]. Associations with HIV RNA viral load are less clear. Although some studies have reported that high HIV RNA viral loads are associated with increased anal cancer risk [7], and viral suppression on antiretroviral therapy (ART) with reduced risk [8], others suggest that these effects might be entirely mediated by CD4 count [6]. A 2020 meta-analysis included 14 studies examining the association between HIV-related factors and anal cancer incidence [8], but none was from sub-Saharan Africa. Although two-thirds of PWH live in sub-Saharan Africa, information about incident anal cancer and markers of HIV disease severity in the region is scarce. To address this gap, we examined the association of CD4 count and HIV RNA viral load with incident anal cancer among PWH in South Africa.

## METHODS

We conducted a cohort study using medical claims from Aid for AIDS (AfA), a private-sector program providing HIV management to individuals enrolled in various medical insurance schemes in South Africa. We included individuals aged ≥18 years who were members of the AfA program between January 1, 2011 and December 1, 2022, and who had at least two CD4 cell count measurements and two HIV RNA viral load measurement during that time. Our primary endpoint was a diagnosis of incident anal cancer, defined as at least two C21 International Classification of Diseases (ICD)-10 codes recorded on separate days. Individuals with one C21 ICD-10 code were excluded from the main analyses but included in a sensitivity analysis. An individual's follow-up time started at the latest of the following time-points: 6 months after their first CD4 count, 6 months after their first HIV RNA viral load measurement, their 18th birthday, or January 1, 2011. It ended at whichever came first from: program exit, December 1, 2022, or anal cancer diagnosis.

We generated descriptive statistics for people with and without an anal cancer diagnosis. We fit Cox proportional hazards models to assess the association between CD4 count, HIV RNA viral load, and the risk of anal cancer. CD4 count and HIV RNA viral load measurements were lagged by 6 months, and time-updated on a month-by-month basis using linear interpolation. We derived hazard ratios (HR) for the association between CD4 count, HIV RNA viral load, and anal cancer from (1) unadjusted models for CD4 count and HIV RNA viral load; (2) partially adjusted models for CD4 count and HIV RNA viral load, controlling for sex, age at start of follow-up, calendar year at start of follow-up, and ART use (no/yes, time-updated); and (3) a fully adjusted model including both CD4 count and HIV RNA viral load, and controlling for the covariates from the previous model. We considered CD4 count and HIV RNA viral load as either categorical variables (categories: <200, 200–499, ≥500 cells/μL, and <1000 and ≥1000 copies/mL, respectively) or continuous variables (per 100 cells/μL decrease and per log10 copies/mL unit increase, respectively). HIV RNA viral loads below the detection limit were set to 20 copies/mL.

## RESULTS

Of 171 374 people enrolled in the AfA program, we included data on 130 992 individuals (58% women). Reasons for exclusions are presented in Supplementary Figure 1. Median age at the start of follow-up was 42.6 years (interquartile range [IQR] 38.0–50.3) for PWH who developed anal cancer, and 39.0 years (IQR 33.2–46.0) for those who did not (Supplementary Table 1). Most individuals (96%) initiated ART before the end of their follow-up time. Over 768 157 person-years (median follow-up time per individual: 5.5 years, IQR 2.5–9.0), 60 incident anal cancers were diagnosed, resulting in an incidence rate of 7.8 per 100 000 person-years (95% confidence intervals [CI] 6.0–10.1). At the start of follow-up, individuals who later developed incident anal cancer had lower CD4 counts and higher HIV RNA viral loads than individuals who did not develop anal cancer (Supplementary Table 1).

**Figure 1.**
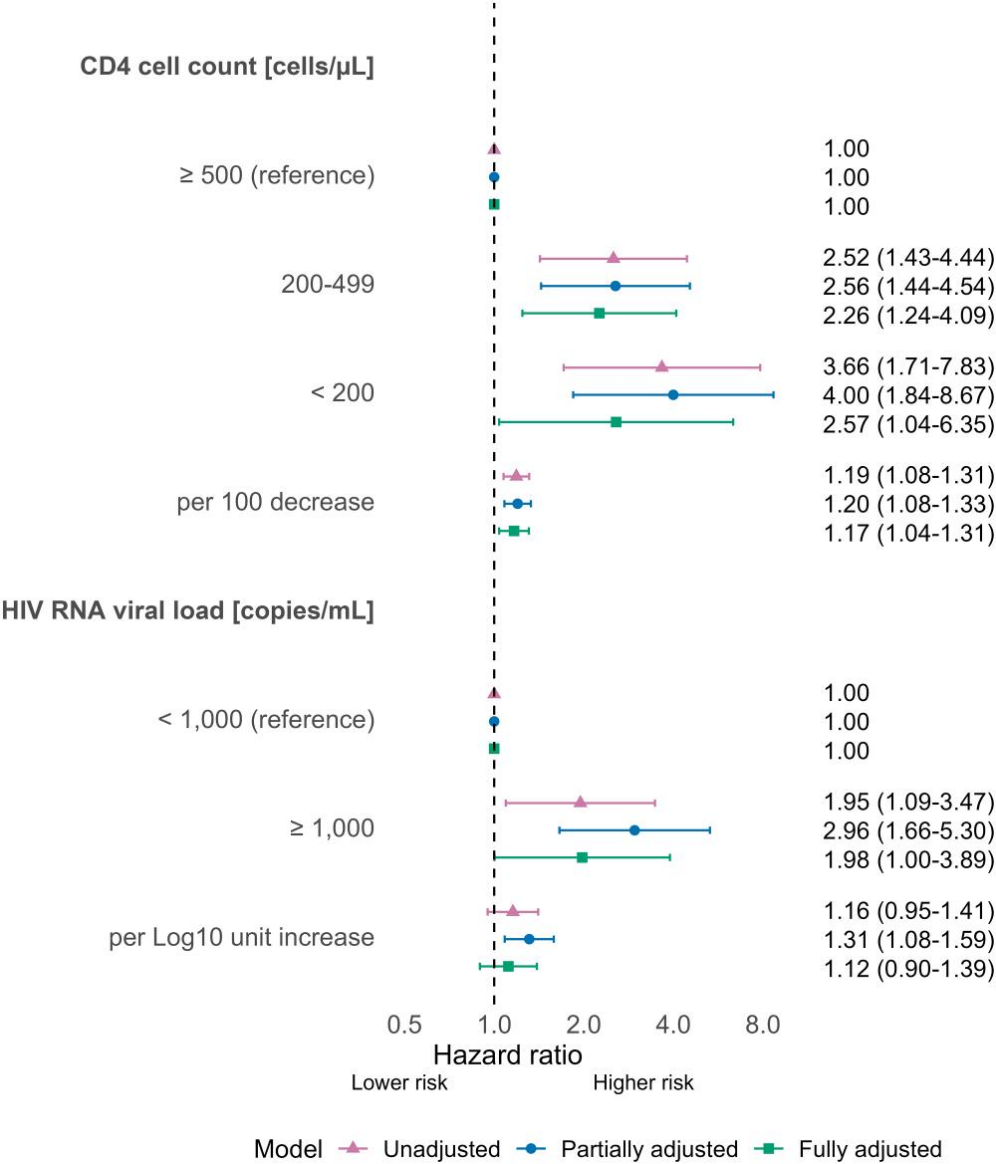
Hazard ratios and 95% confidence intervals for the association of CD4 cell count and HIV RNA viral load with incident anal cancer diagnosis. The estimates for the categorical and continuous variables are derived from separate models. Partially adjusted models control for sex, age, calendar year, and ART status. Fully adjusted models also control for the other marker (CD4 cell count or HIV RNA viral load).

Lower CD4 counts were associated with an increased anal cancer risk in all models (Figure 1). The rate of anal cancer increased by 17% (HR: 1.17, 95% CI 1.04–1.31) per 100 CD4 cells/µL decrease, after controlling for HIV RNA viral load and the other factors. A 10-fold increase in HIV RNA viral load was associated with a higher risk of anal cancer (HR 1.31; 95% CI 1.08–1.59) in the partially adjusted model but the HR decreased to 1.12 (95% CI 0.90–1.39) after further controlling for CD4 count. There was some evidence of a higher risk of anal cancer among individuals with HIV RNA viral loads ≥1000 copies/mL than those with suppressed viral load (adjusted HR 1.98, 95% CI 1.00–3.89), after controlling for CD4 count. When we included individuals with a single C21 ICD-10 code as patients with anal cancer, the association with CD4 count and HIV RNA viral load was similar to the main analysis (Supplementary Figure 2).

## DISCUSSION

We found that lower CD4 counts and, to a lesser extent, higher HIV RNA viral loads were associated with an increased risk of anal cancer diagnosis in this private-sector HIV disease management program. While elevated anal cancer rates among PWH with low CD4 cell counts are well documented, the association between HIV RNA viral load and anal cancer risk is less well studied [6–8]. A French study found that both prolonged periods of CD4 cell counts <200 cells/μL and HIV RNA viral loads >100 000 copies/mL were associated with an increased anal cancer risk in mutually adjusted analyses [7], pointing toward a possible independent role of cellular immunodeficiency and viremia-induced immune activation in anal carcinogenesis. In contrast, a North American study found that while nadir and cumulative CD4 cell counts were strong predictors of anal cancer risk, adding HIV RNA viral load did not further improve the prediction [6]. This supports the hypothesis that the link between HIV RNA viral load and anal cancer risk may be mostly mediated by HIV-induced immunodeficiency.

International guidelines recommend screening for anal dysplasia among different population groups at increased risk for anal cancer, particularly among PWH [9]. Beyond routine anal cancer screening, close monitoring of CD4 counts could help identify PWH at heightened risk of anal cancer, enabling targeted screening and prevention strategies. This emphasizes the importance of obtaining a CD4 count at the time of HIV diagnosis and monitoring it until sustained virologic suppression and CD4 recovery. Yet, this practice has been declining in Southern Africa over time with the adoption of the “Treat All” ART policy [10]. Human papillomavirus vaccines have shown high efficacy in preventing anal hrHPV infection when administrated to anal hrHPV–naïve individuals [11]. Universal, gender-neutral HPV vaccination strategies—particularly among children and adolescents—could substantially reduce the burden of anal cancer in the future [11].

Our study of more than 130 000 PWH is among the first to examine the association of HIV disease severity markers and anal cancer risk among PWH in sub-Saharan Africa. However, our study has some limitations. We used medical claims data to define anal cancer diagnoses, which may have led to some misclassification and underreporting. To reduce the likelihood of false-positive anal cancer diagnoses, we required at least two C21 ICD-10 codes for diagnosis and assessed the impact of changing our anal cancer definition in a sensitivity analysis. Results of the sensitivity analysis showed a similar pattern. Moreover, data on other biomarkers of interest such as the CD4/CD8 ratio [12] and potential confounders such as smoking and sexual orientation were unavailable. Furthermore, we could not stratify our analyses by anal cancer morphology as histological information was mostly missing. The sociodemographic characteristics and health care access of our study population differ from PWH receiving care in South Africa's public sector or other countries, which may limit the generalizability of our findings and partly explain the lower anal cancer rate observed compared with studies from the US and Europe [2, 7]. However, while absolute risk estimates may not be generalizable, relative associations between HIV-related factors and incident anal cancer are likely more robust, as they reflect internal comparisons.

In conclusion, lower CD4 counts were associated with an increased risk of anal cancer diagnosis. These findings suggest that maintaining high CD4 counts may contribute to anal cancer prevention among PWH in South Africa.

## Supplementary Material

ofaf693_Supplementary_Data

## Supplementary Data

Supplementary materials are available at *Open Forum Infectious Diseases* online. Consisting of data provided by the authors to benefit the reader, the posted materials are not copyedited and are the sole responsibility of the authors, so questions or comments should be addressed to the corresponding author.

## References

[1] Silverberg MJ, Lau B, Justice AC, et al Risk of anal cancer in HIV-infected and HIV-uninfected individuals in North America. Clin Infect Dis 2012; 54:1026–34. 22291097 10.1093/cid/cir1012 PMC3297645

[2] Colón-López V, Shiels MS, Machin M, et al Anal cancer risk among people with HIV infection in the United States. J Clin Oncol 2018; 36:68–75. 29140774 10.1200/JCO.2017.74.9291 PMC5791846

[3] Lin C, Franceschi S, Clifford GM. Human papillomavirus types from infection to cancer in the anus, according to sex and HIV status: a systematic review and meta-analysis. Lancet Infect Dis 2018; 18:198–206. 29158102 10.1016/S1473-3099(17)30653-9 PMC5805865

[4] Cachay ER, Gilbert T, Qin H, et al Clinical predictors and outcomes of invasive anal cancer for people with HIV in an inception cohort. Clin Infect Dis 2024; 79:709–16. 38573010 10.1093/cid/ciae124 PMC11426273

[5] Llibre JM, Revollo B, Aceiton J, et al Identifying risk factors for anal cancer in people with HIV in Spain: a multicentre retrospective cohort study nested in the PISCIS cohort. Lancet HIV 2024; 11:e598–606. 39102835 10.1016/S2352-3018(24)00174-7

[6] Hernández-Ramírez RU, Qin L, Lin H, et al Association of immunosuppression and human immunodeficiency virus (HIV) viremia with anal cancer risk in persons living with HIV in the United States and Canada. Clin Infect Dis 2020; 70:1176–85. 31044245 10.1093/cid/ciz329 PMC7319056

[7] Guiguet M, Boué F, Cadranel J, et al Effect of immunodeficiency, HIV viral load, and antiretroviral therapy on the risk of individual malignancies (FHDH-ANRS CO4): a prospective cohort study. Lancet Oncol 2009; 10:1152–9. 19818686 10.1016/S1470-2045(09)70282-7

[8] Kelly H, Chikandiwa A, Alemany Vilches L, et al Association of antiretroviral therapy with anal high-risk human papillomavirus, anal intraepithelial neoplasia, and anal cancer in people living with HIV: a systematic review and meta-analysis. Lancet HIV 2020; 7:e262–78. 32109408 10.1016/S2352-3018(19)30434-5

[9] Stier EA, Clarke MA, Deshmukh AA, et al International anal neoplasia Society's consensus guidelines for anal cancer screening. Int J Cancer 2024; 154:1694–702. 38297406 10.1002/ijc.34850

[10] Zaniewski E, Brazier E, Ostinelli CHD, et al Regression discontinuity analysis demonstrated varied effect of treat-all on CD4 testing among Southern African countries. J Clin Epidemiol 2021; 140:101–10. 34487837 10.1016/j.jclinepi.2021.09.001 PMC8712349

[11] Barroso LF, Stier EA, Hillman R, et al Anal cancer screening and prevention: summary of evidence reviewed for the 2021 centers for disease control and prevention sexually transmitted infection guidelines. Clin Infect Dis 2022; 74:S179–92. 35416975 10.1093/cid/ciac044

[12] Stem J, Hewitt AJ, Yang Q, et al Commonly drawn immunologic and inflammatory markers as risk predictors for anal cancer in veterans living with HIV. J Low Genit Tract Dis 2024; 28:300–4. 38661377 10.1097/LGT.0000000000000811 PMC11213675

