# Supplementary material for: Immunodeficiency, HIV Viremia, and Incident Anal Cancer Among People With HIV in South Africa
Source: Open Forum Infect Dis. 2025 Nov 12;12(12):ofaf693. doi: 10.1093/ofid/ofaf693 (PMC12651545; doi:10.1093/ofid/ofaf693)
Supplement: ofaf693_Supplementary_Data [file ofaf693_supplementary_data.docx]

**Supplementary Material:**

**Immunodeficiency, HIV viremia, and incident anal cancer among people with HIV in South Africa**

**Supplementary Table 1:** Study population characteristics stratified by anal cancer diagnosis

|  | **No anal cancer**  **(N = 130,932)** | **Anal cancer**  **(N = 60)** |
| --- | --- | --- |
| **Female, n (%)** | 76,300 (58.3) | 29 (48.3) |
| **Median age* [IQR]** | 39.0 [33.2 - 46.0] | 42.6 [38.0 - 50.3] |
| **Calendar year*, n (%)** |  |  |
| 2011-2013 | 59,157 (45.2) | 33 (55.0) |
| 2014-2016 | 32,411 (24.8) | 18 (30.0) |
| 2017-2019 | 25,943 (19.8) | 7 (11.7) |
| 2020-2022 | 13,421 (10.3) | 2 (3.3) |
| **CD4 cell count [cell/µL]*, n (%)** |  |  |
| 0-99 | 14,551 (11.1) | 15 (25.0) |
| 100-199 | 17,475 (13.3) | 12 (20.0) |
| 200-349 | 30,214 (23.1) | 16 (26.7) |
| 350-499 | 25,309 (19.3) | 9 (15.0) |
| ≥500 | 43,383 (33.1) | 8 (13.3) |
| Median [IQR] | 366 [203 - 579] | 239 [112 - 370] |
| **HIV RNA viral load [copies/mL]*, n (%)** |  |  |
| 0-49 | 50,048 (38.2) | 18 (30.0) |
| 50-499 | 13,015 (9.9) | 7 (11.7) |
| 500-999 | 16,591 (12.7) | 9 (15.0) |
| 1,000-99,999 | 27,188 (20.8) | 14 (23.3) |
| ≥100,000 | 24,090 (18.4) | 12 (20.0) |
| Median [IQR] | 903 [20 - 53,139] | 3,531 [20 - 76,209] |
| **Initiated ART**, n (%)** | 126,137 (96.3) | 59 (98.3) |

ART: Antiretroviral therapy, IQR: Interquartile range

*At start of follow-up time, **During or before follow-up time


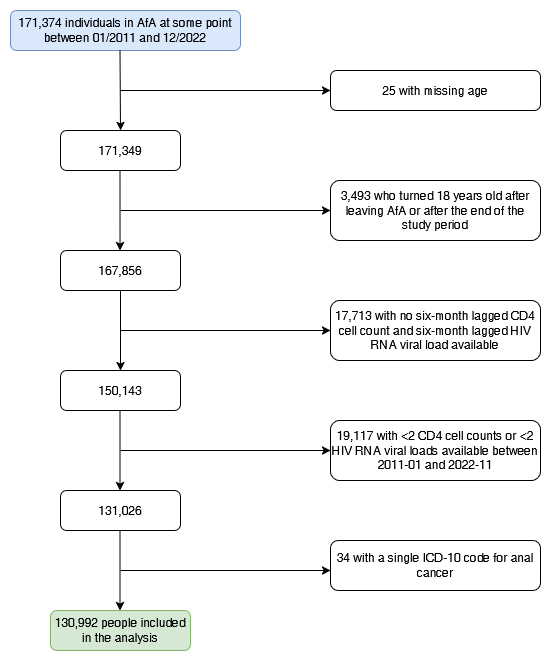


**Supplementary Figure 1:** Selection of study population.

**Alt text supplementary figure 1:** Flow diagram of study population selection with boxes showing numbers excluded for specific reasons and arrows leading to the final sample included in the analysis.

**
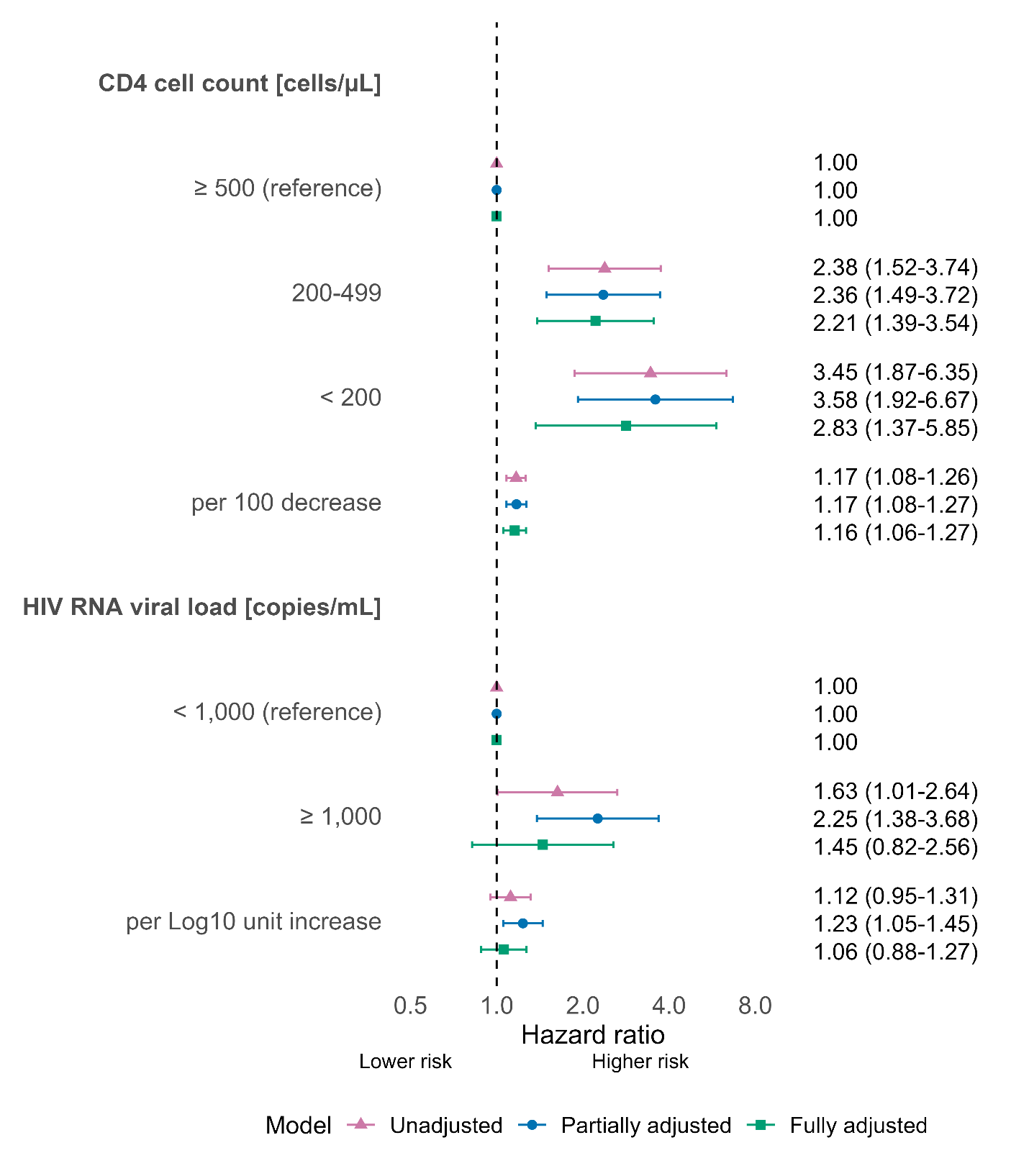
**

**Supplementary Figure 2:** Hazard ratios and 95% confidence intervals for the association of CD4 cell count and HIV RNA viral load with incident anal cancer, including incident anal cancer diagnoses with a single ICD-10 code. The estimates for the categorical and continuous variables are derived from separate models. Partially adjusted models control for sex, age, calendar year, and antiretroviral therapy status. Fully adjusted models also control for the other marker (CD4 cell count or HIV RNA viral load).

**Alt text supplementary figure 2:** Forest plot of hazard ratios with 95% confidence intervals for the association of CD4 cell count and HIV RNA viral load with incident anal cancer when including incident anal cancer diagnoses with a single ICD-10 code. Estimates from both categorical and continuous variable representations are presented.
